# Management of post-infection forearm defect non-unions following the “Road-to-Union” protocol: technical note and case series

**DOI:** 10.1007/s00590-026-04663-8

**Published:** 2026-02-02

**Authors:** Franz Friedrich Birkholtz, Annette-Christi Barnard, Maaike Maria Eken, Festus Iiyambula, Peter O’Farrell

**Affiliations:** 1 Institute of Orthopaedics and Rheumatology, Mediclinic Winelands Orthopaedic Hospital, Stellenbosch, South Africa; 2 Division of Orthopaedic Surgery, Department of Surgical Sciences, Faculty of Medicine and Health Sciences, Tygerberg, South Africa; 3 Rehab at Muelmed, Rehabilitation Spinal and Neuro Unit, Muelmed Mediclinic, Pretoria, South Africa; 4 Division of Sport and Exercise Medicine, Department of Exercise, Sport and Lifestyle Medicine, Faculty of Medicine and Health Sciences, Stellenbosch University, Tygerberg, South Africa; 5 Lower Limb Trauma and Limb Reconstruction, Department of Orthopaedic Surgery, Steve Biko Academic Hospital, Pretoria, South Africa; 6 Upper Limb and Hand Surgery, Mediclinic Worcester, Worcester, South Africa

**Keywords:** Distraction osteogenesis, Limb salvage, External fixators, Forearm

## Abstract

Diaphyseal non-union of forearm fractures that involve both the ulna and radius present unique challenges to treat. If left untreated, these non-unions may lead to severe instability of the forearm and/or chronic pain. Existing treatments include bone grafting, fibular grafts, and the Masquelet technique, however, currently no gold standard treatment exists. The “Road-to-Union” protocol is a two-stage surgical approach originally developed for managing complex tibial bone defects. It integrates debridement, circular external fixation, soft-tissue management, distraction osteogenesis, and structured rehabilitation. This technique addresses challenges such as infection, bone loss, and deformity by providing a systematic pathway to achieve bone healing and restore function. While traditionally used in the lower limb, its application in the forearm has not been widely reported. This case series explores the adaptation of the “Road-to-Union” protocol for forearm reconstruction, aiming to restore structural integrity and function in complex non-union cases.

## Introduction

Non-union of forearm fractures, particularly those involving both the ulna and radius, may substantially impact a patient’s function and overall well-being. Early studies have shown that infection after fracture fixation can occur in 1–2% of closed fractures and up to 30% of open fractures [1–3]. In diaphyseal forearm fractures specifically, infection rates of 2–6% have been reported [4, 5], with one study showing an infection rate of 31% (11/35 patients) [6]. The combination of complex musculoskeletal function in the forearm, along with factors such as infection, sclerotic bone ends, large bone defects, shortening and deformities present great challenges to orthopaedic surgeons in providing optimal treatment to regain full forearm function [7].

Different techniques have been developed to treat these complex conditions, including the one-bone technique [8], cortico-cancellous bone graft/interposition grafting, double-barrel free fibula reconstruction [9], vascularised and non-vascularised fibular graft, Masquelet’s induced membrane technique [10], titanium cage reconstruction and bone transport. Despite the range of available options, limited research is available showing the effectiveness of each technique. As a result, none of these techniques have emerged as a definitive gold standard for the treatment of these intricate fractures.

The ‘Road-to-Union’ protocol describes a two-stage surgical strategy that integrates different methods to treat tibial bone defects [11]. This stepwise care pathway has shown high union rates in cases of complex tibial trauma and bone defects [12]. The core principle of the protocol is to restore the continuity of the bone, which in turn allows for restoration of the bone’s role within the limb and overall limb function. This principle applies whether the bone is for weight bearing and locomotion, as in the lower leg, or for range of motion and fine motor movements, as in the forearm. Preliminary evidence from a case report supports the premise that the protocol is applicable to forearm bone defects [13]. Notably, it allows for independent reconstruction of the radius and ulna, preserving forearm length and avoiding the biomechanical sequelae of shortening large defects. The current study builds on the initial case study, by providing a technical description of the “Road-to-Union” protocol to treat diaphyseal forearm bone defects, involving both the radius and ulna, along with outcomes from the first three cases.

## Procedure

The full “Road-to-Union” protocol consists of seven stages:

Step 1: *Debridement*,* PMMA spacer using the Masquelet technique*,* and provisional stabilisation with external fixation*.

In case patients present with existing implants, they are to be removed, and non-viable bone and fibrotic tissue are excised until healthy, bleeding bone is encountered [14–16]. Following debridement, wounds are irrigated with copious amounts of Prontosan Wound Irrigation Solution (B.Braun Medical Inc) and saline. Bone fragments are stabilised using rail fixators, e.g. paediatric Limb Reconstruction System (LRS) rail fixators (Orthofix, Verona, Italy). The radius and ulna are then temporarily stabilized with separate fixators anchored to the bone using hydroxy-apatite conical half pins.

Once the defect size is determined, an antibiotic loaded polymethyl methacrylate (PMMA) spacer is provisionally shaped outside the body to limit thermal damage and inserted just before curing to allow for modifications of the spacer to achieve overlap with bone ends [11]. The radius and ulna are treated independently to maintain anatomical orientation and permit future bone transport.

Step 2: *Soft tissue coverage and wound closure*.

**Fig. 1:**
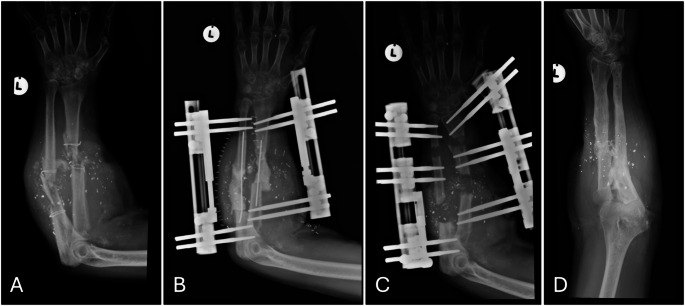
**A**–**D**. Radiographs from case 3: **A** Radiograph showing bullet fragments and cerclage wires in situ, and a comminuted diaphyseal fracture previously managed surgically; **B** Post initial debridement radiograph showing spacer in situ, held in place with intramedullary wires, and fractures stabilised with LRS fixators separately for radius and ulna bones; **C** Post corticotomy radiograph with proximal to distal bone transport showing early regenerate formation; **D** Radiograph at final follow up demonstrating full consolidation of regenerate bone

With the PMMA spacer in place (Fig. 1B), primary closure of the incision site is attempted, taking care to close the skin under minimal tension. This will be facilitated by overall shortening of the affected forearm compared to the contralateral side, which not only facilitates primary closure but also reduces the size of the primary bone defect.

Step 3: *PMMA spacer removal and corticotomy*.

In case of a resistant infection, the fracture site may need to be debrided multiple times, which includes the exchanges of the PMMA spacer to eradicate the infection. The patient is then converted to culture-specific antibiotics and needs to start functional hand rehabilitation.

After 6–10 weeks, once the soft tissue envelope has matured and the induced membrane has formed, the patient will be asked to return to theatre for spacer removal and corticotomy.

A minimal longitudinal incision is then made through the previous scar to remove the spacer. The induced membrane is closed with a resorbable suture, followed by a low energy predrilled corticotomy through a 1 cm incision in the meta-diaphyseal bone to prepare for bone transport [17]. The periosteum will be left intact as far as possible to encourage regenerate formation (Fig. 1C).

Step 4: *Latency period and gradual distraction*.

Distraction commences after a latency period of 10 days at a rate of 1 mm per day in increments of 0.25 mm, allowing for osteogenesis in the created gap. Adjustment of the distraction rate is based on clinical and radiographic findings.

Step 5: *Docking site modification*.

Once the transport fragment is in proximity of less than 1 cm to the target fragment, the patient will undergo a second surgery for open docking and docking site modification. Both bone ends will be surgically exposed, debrided until bleeding bone, and any sclerotic edges resected. The medullary canals will be re-canalized using a 3.5 mm drill bit to encourage vascular communication. After modification of the docking site, acute compression of the fragments is achieved using the external fixator, under image intensifier guidance.

If there is a small distal fragment and the transport fragment can not be approximated to the distal fragment, an interposition autograft can be used to bridge the gap.

Step 6: *Functional rehabilitation*.

Occupational therapy will be initiated within 14 days post-debridement to minimize stiffness and preserve hand and elbow function. Therapy will include active-assisted range-of-motion exercises, edema control, and progressive strengthening. Rehabilitation continues for four weeks, with patients educated on continued home exercise programs.

Step 7: *Frame removal and long-term surveillance*.

The patient will be clinically and radiologically followed up twice a month during the transport phase and monthly during the consolidation phase. Once union at the docking site is established and the regenerate is consolidated (Fig. 1D), the external fixator will be removed. A circular cast will be applied for 4 weeks, followed by transition to a removable splint for an additional 4 weeks.

## Case series

This case series comprises three patients with bone defects in the forearm following fractures. All patients were managed by a single surgeon (FB) at a specialised limb lengthening centre between 2009 and 2016. A description of one case has been published previously, with reproduction of the information provided for by the journal’s unrestricted use open access policy [13]. Brief clinical and demographic characteristics are presented in Table 1. Ethical approval for this case series was provided by Stellenbosch University (C24/12/035). All patients signed informed consent for their anonymised data to be used in a case report.

**Fig. 2:**
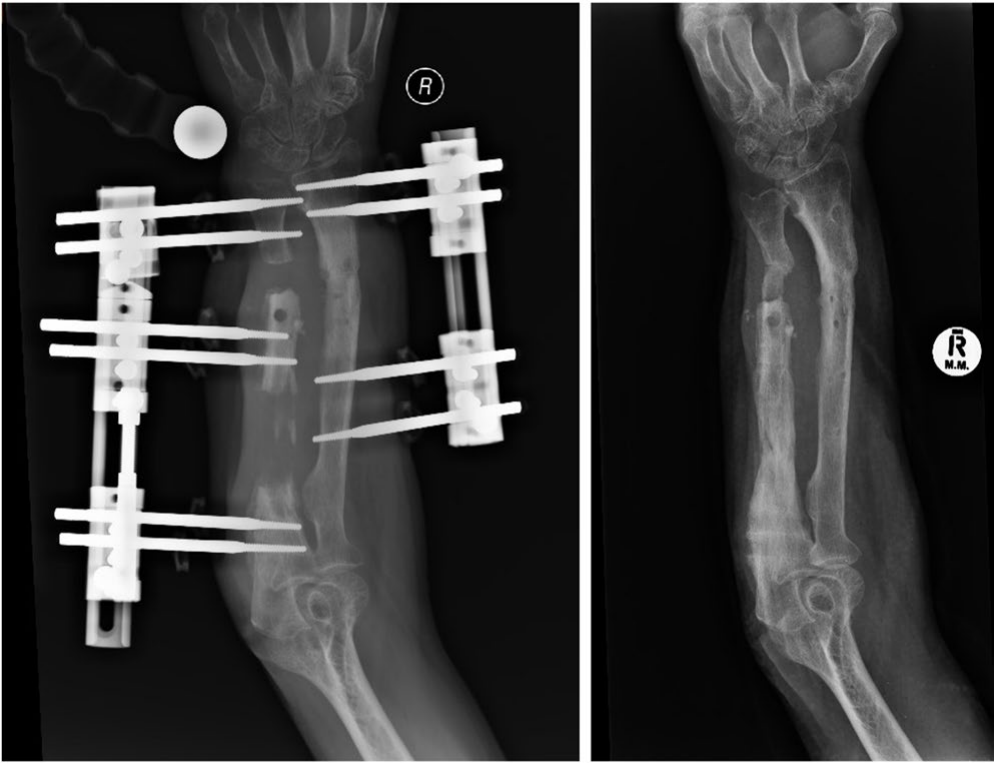
Distraction and outcome radiographs, Case 1

**Fig. 3:**
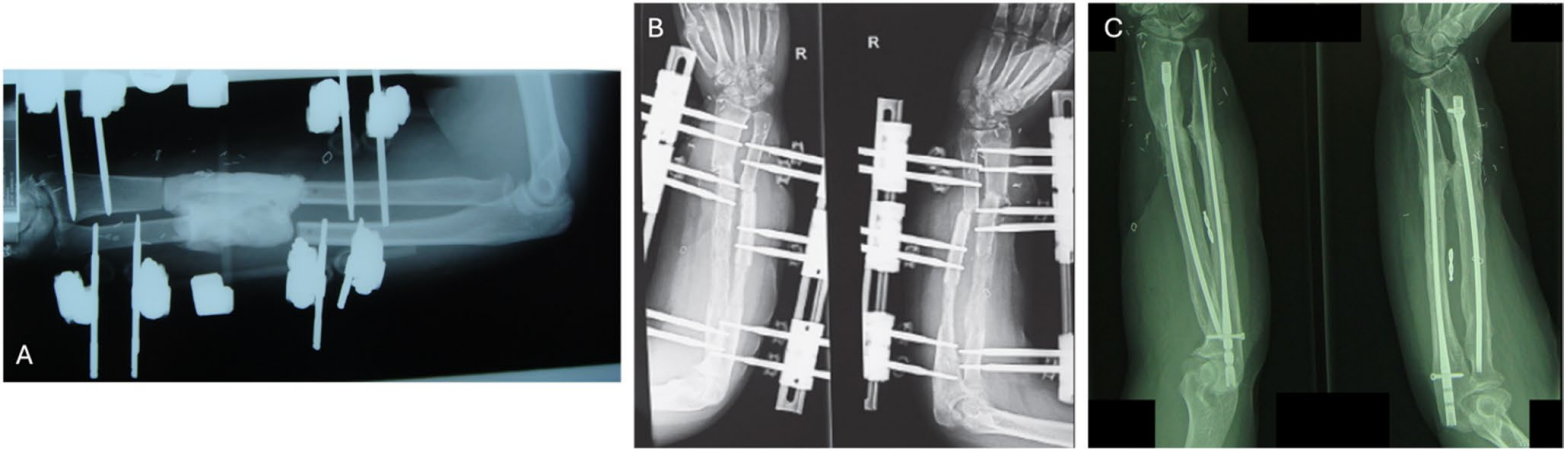
Radiographs for Case 2 **A** before distraction, **B** during distraction, and **C** at final outcome, with internal nails. **A**, **B** have been published previously [13]

**Fig. 4:**
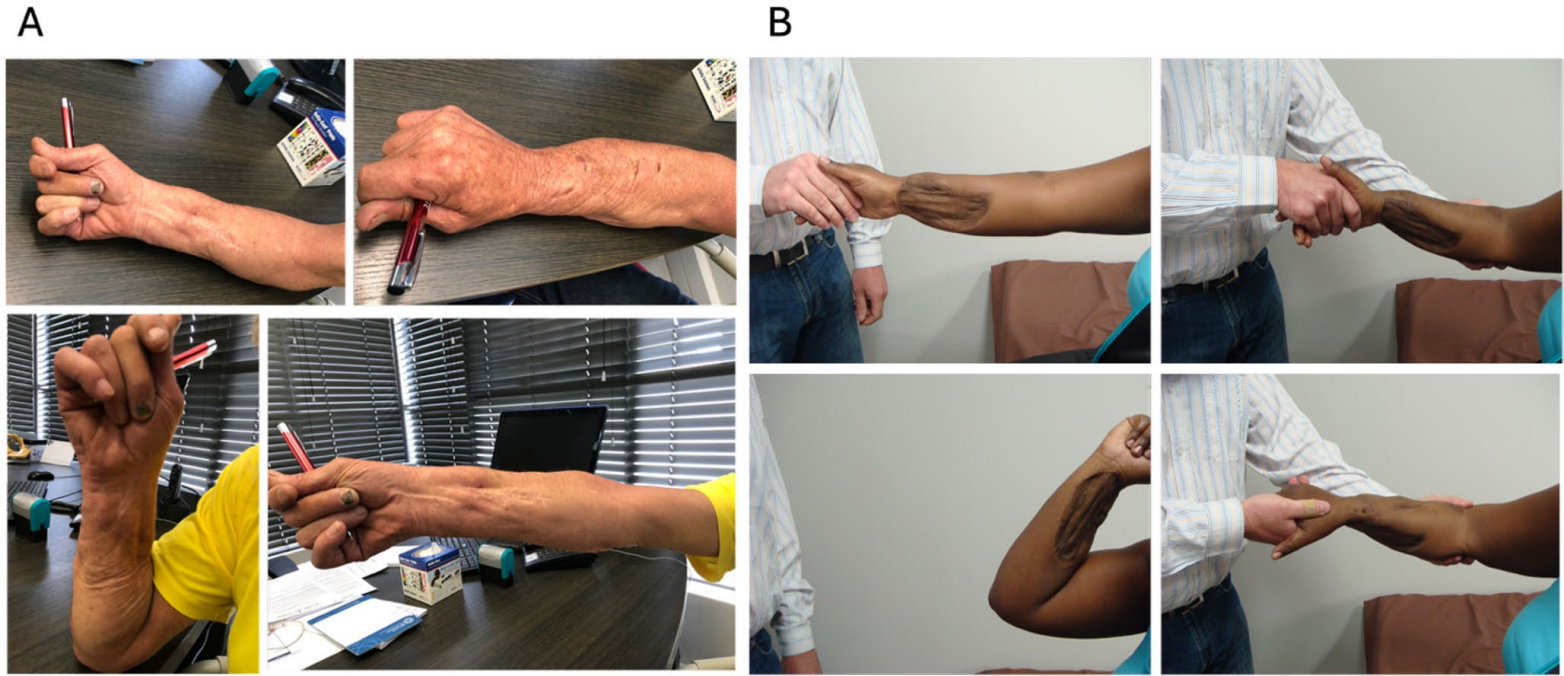
Range of motion at final follow-up for **A** Case 1, and **B** Case 2. **B** has been published previously [13].

Treatment outcomes including pain and function at final follow-up are shown in Table 1. Radiographs for Cases 1 and 2 are shown in Figs. 2 and 3. Final range of motion for Cases 1 and 2 is shown in Fig. 4. Union was achieved in all three patients and none showed clinical signs of infection. Furthermore, none showed clinical or radiological synostosis. Cases 1 and 3 required no permanent implant following treatment whereas, in Case 2, conversion to intramedullary nailing was performed to shorten the external fixation time.

**Table 1:** Outcome measures at the last follow-up

	Case 1	Case 2	Case 3
Age (years)	58	34	43
Sex	Male	Female	Male
Affected side	Right, dominant	Right, dominant	Left, non-dominant
Previous surgery	Yes	Yes	Yes
Initial cause of fracture	Open both bone forearm fracture while on duty	Motor vehicle accident with open comminuted both bone forearm fracture	Gunshot in forearm
Radius bone defect (cm)	2	5	6
Ulna bone defect (cm)	7	5	4
Days in external fixator	194	140	64
Union (Y/N)	Y	Y	Y
Pain score (0–10)	3	0	3
Quick DASH (0–100)	25.0	22.7	18.2
Patient satisfaction (0–10)	10	10	5
Use of pain medication (Y/N)	N	N	N
Return to former occupation (Y/N)	N	N	Y
Functional capability ^a^	Activities of daily living	Activities of daily living	Activities of daily living
Loss of ROM elbow (°)	< 10°	< 10°	< 10°
Loss of ROM wrist (°)	< 10°	< 10°	< 10°
Loss of ROM forearm rotation (°)	< 25°	< 25°	< 25°
ROM test result^b^	Excellent outcome	Excellent outcome	Excellent outcome

^a^Functional capability level was classified into: Near normal, complex activities, activities of daily living or minimal [18]

^b^ROM was classified as follows: a healed fracture with < 10° loss of elbow or wrist motion and < 25% loss of forearm rotation as excellent, a healed fracture with < 20° loss of elbow or wrist motion and < 50% loss of forearm rotation as satisfactory, a healed fracture with more than 30° loss of elbow or wrist motion and more than 50% loss of forearm rotation as unsatisfactory, and a malunion, non-union, or unresolved chronic osteomyelitis as failure [6]

## Discussion

The “Road-to-Union” protocol has previously been shown to be effective in the treatment of complex tibial fractures [11]; this case series demonstrates that it also resulted in union of post-infection forearm defect non-unions. Notably, the protocol leads to largely favourable outcomes regarding arm function and rotation, which are particular challenges in the management of complex diaphyseal forearm non-unions.

Various techniques have been developed over recent years to manage defect non-unions of diaphyseal forearm fractures. Interestingly, a recent scoping review indicated that the majority of previous cases involving both ulna and radius forearm fractures have been treated with the one-bone forearm technique (69% of both forearm fractures) [19]. The fusion of radius and ulna effectively converts the two-bone forearm structure into a single-bone structure, which can enhance stability and promote healing in cases where traditional methods have failed [8]. However, fixation of the anatomical relationship between the radius and ulna can restrict pronation and supination function [20, 21], significantly impacting the patient’s range of motion. Loss of motion in the forearm has been reported to lead to limited quality of life in patients with malunion of both bones of the forearm [22].

In contrast to the one-bone technique, other techniques have aimed to facilitate independent bone union and restore the relationship between the radius and ulna. Single stage plate fixation with autologous bone graft has often been used [23, 24], however, poor outcome have been reported when defects were larger than 5 cm, owing to a high likelihood of graft resorption and weakening at the graft site [25–27]. Studies using the Masquelet technique, as used in the “Road-to-Union” protocol, have shown its effectiveness in treatment of long bone defects of the lower limb [28–32], while fewer studies have reported on its use in the upper limb [33, 34]. However, when the Masquelet technique is used as a stand-alone treatment, it has been reported that bone integration is slow and that mechanical failure may occur [35]. In addition, previous research showed superior clinical recovery rates and bone healing indices in patients who had undergone bone transport techniques compared to the use of the Masquelet technique [36]. Reported advantages included faster overall healing time, fewer complications and improved lower limb function.

To overcome shortcomings of the different techniques, the “Road-to-Union” protocol combines the Masquelet technique with bone transport, which has proven successful in achieving union in large bone defects of complex tibial fractures [11, 12]. In the current case series, bone transport was initiated after a latency period of 10 days, slightly longer than reported by Hohmann et al. (2017) in complex tibial fractures [11]. However, patients in the current case series experienced minimal loss of range of motion in the elbow, wrist, and forearm, with motion in the forearm being the most limited. All three cases involved distraction osteogenesis in the proximal radius, a relatively straight segment of bone, and this may have contributed to the favourable functional outcomes. Slight angulation of the external fixator pins in the distal radius segment was also used in an attempt to maintain some radius curvature. It is unclear whether distraction osteogenesis in a segment that altered the radius curvature would retain similar functionality.

It is important to note that the “Road-to-Union” protocol for infected non-union of forearm fractures requires highly advanced surgical skills. Previous research has shown that when patients undergo surgery for forearm fractures, adverse events are commonly reported (31%), including major adverse events (18%) [37]. Therefore, it is recommended that surgeries for complex forearm fractures should be performed by experienced surgeons, restricting inexperienced surgeons from operating on these fractures without supervision.

## Conclusion

This case series demonstrated that the Road-to-Union protocol, which was originally developed for tibial bone defects, can successfully treat diaphyseal forearm bone defects involving the radius and ulna. By independently restoring the continuity of each bone, the protocol promoted restoration of the forearm anatomy, and only modest loss of function was observed. While these early results are encouraging, more cases, and variation in bone defect location are needed to strengthen understanding of the protocol’s effectiveness. Given its technical complexity, implementation of the Road-to-Union protocol for forearm bone defects should be led by surgeons with advanced reconstruction expertise.

## Data Availability

No datasets were generated or analysed during the current study.
